# Risk Factors for Severe Pain and Impairment of Daily Life Activities after Cesarean Section—A Prospective Multi-Center Study of 11,932 Patients

**DOI:** 10.3390/jcm12226999

**Published:** 2023-11-09

**Authors:** Norah L. A. Emrich, Laura Tascón Padrón, Marcus Komann, Christin Arnold, Johannes Dreiling, Winfried Meißner, Brigitte Strizek, Ulrich Gembruch, Jorge Jiménez Cruz

**Affiliations:** 1 Department of Obstetrics and Prenatal Medicine, University Hospital Bonn, Venusberg-Campus 1, 53127 Bonn, Germany; laura.tascon@ukbonn.de (L.T.P.); brigitte.strizek@ukbonn.de (B.S.); ulrich.gembruch@ukbonn.de (U.G.); jorge.jimenez_cruz@ukbonn.de (J.J.C.); 2 Department for Anesthesiology and Intensive Care Medicine, University Hospital of Jena, Am Klinikum 1, 07740 Jena, Germany; marcus.komann@med.uni-jena.de (M.K.); christin.arnold@med.uni-jena.de (C.A.); johannes.dreiling@med.uni-jena.de (J.D.); winfried.meissner@med.uni-jena.de (W.M.)

**Keywords:** postoperative pain, cesarean section, analgesic treatment, non-pharmacological therapy, primary cesarean section, urgent cesarean section, opioids

## Abstract

Cesarean section (CS) is the most widely performed and one of the most painful surgeries. This study investigated postoperative pain after CS using patient-related outcomes (PROs) to identify risk factors for severe pain. The secondary outcome was to evaluate the influence of surgery indication (primary CS (PCS) vs. urgent CS (UCS)). This multi-center, prospective cohort study included data submitted to the pain registry “quality improvement in postoperative pain treatment” (QUIPS) between 2010 and 2020. In total, 11,932 patients were evaluated. Median of maximal pain was 7.0 (numeric rating scale (NRS) 0 to 10); 53.9% suffered from severe pain (NRS ≥ 7), this being related to impairment of mood, ambulation, deep breathing and sleep, as well as more vertigo, nausea and tiredness (*p* < 0.001). Distraction, relaxation, mobilization, having conversations, patient-controlled analgesia (PCA) and pain monitoring were shown to be protective for severe pain (*p* < 0.001). Maximal pain in PCS and UCS was similar, but UCS obtained more analgesics (*p* < 0.001), and experienced more impairment of ambulation (*p* < 0.001) and deep breathing (*p* < 0.05). Severe pain has a major effect on daily-life activities and recovery after CS, and depends on modifiable factors. More effort is needed to improve the quality of care after CS.

## 1. Introduction

Cesarean section (CS) is the most painful surgery in gynecology and obstetrics [1]. It is ranked as the 9th most painful surgery out of 179 different analyzed procedures [2]. Inadequately controlled pain after CS is of international concern [3]. A negative effect of insufficient pain relief on patient recovery and quality of life has been demonstrated [4,5]. High postoperative pain levels impair not only the patient’s well-being, but can also have a negative impact on neonatal care, including breastfeeding [6]. Furthermore, severe pain after CS can lead to persistent postsurgical pain, presumably by manifestation of neuropathic pain caused by damage to peripheral nerves. Chronic pain has been demonstrated to be a risk factor for developing depression [7,8,9]. Besides those pain-related impairments to an individual patient, the healthcare system is also being compromised by inadequate analgesic therapy. Prolonged hospitalization or the readmission of women after cesarean delivery due to severe pain leads to major expenditures for health services [10,11,12,13]. From a sociocultural point of view, giving birth is deeply linked to enduring high levels of pain, since delivery pain is considered to be a natural phenomenon [14,15,16,17]. Interestingly, analgesic therapy in women is presumed to be more complex than in men. Tighe et al., in a retrospective cohort study including over 300,000 patients undergoing non ambulatory surgery, found that female patients reported significantly more postoperative severe pain events than males, with an odds ratio (OR) of 1.14 (99% confidence interval (CI) 1.10–1.19) [18]. Furthermore, women have a higher risk of developing persistent postsurgical pain [19,20]. Different effects of pain medication on nociception between women and men due to different pharmacokinetics and dynamics have been proven [21,22,23], with greater morphine efficacy in women [24].

Medical staff as well as patients often hesitate to use opioids after CS because of alleged possible safety impairments for breastfeeding newborns. This could also influence the insufficiency of analgesic therapy after CS [25]. Since CS is the most frequently performed surgery worldwide [26,27,28], the consequences of inadequate postoperative pain relief have a huge impact on the female population.

The aim of this study was to evaluate postoperative pain experience after CS using patient-reported outcomes (PROs) in order to identify risk factors for severe pain, and to detect key points for improving postoperative analgesia in these women. Secondary outcomes were the evaluation of differences regarding pain levels, analgesic therapy and PROs in primary cesarean section (PCS) compared to urgent/emergency cesarean section (UCS).

## 2. Methods

In this multi-center, prospective cohort study, 27 different departments of gynecology and obstetrics all over Germany participated. Data were assembled from 2010 to 2020 in the nationwide pain registry “quality improvement in postoperative pain treatment” (QUIPS). Only departments including at least 200 women within this period of time were included. The 1998 established national registry QUIPS (www.quips-projekt.de, last visited 17 September 2023) is a project for the quality improvement and registration of postoperative pain management using pain-related patient-reported outcomes (PROs) and standard hospital records. One of the goals of QUIPS is self-reflection on the standard procedures of the departments. Additionally, this allows for a comparison between different hospitals through benchmarking, and therefore offers an opportunity to improve pain management, if necessary [29]. To this end, a validated questionnaire was completed by each patient after CS on the first postoperative day 24–32 h after surgery. A trained research assistant not being involved in clinical treatment of any kind visited each patient to receive written consent and to obtain data. The questionnaire comprised 18 questions concerning pain-related PROs and side effects, the utilization of non-pharmacological therapy, satisfaction with analgesic treatment as well as intensity of postoperative pain events measured by an 11-point numeric rating scale (NRS, 0 = no pain, 10 = worst pain imaginable) [30] (Supplementary S1, translated version). Furthermore, data collection regarding planned analgesic treatment, anesthesia, surgery and on-demand medication were registered.

Clinical as well as demographic data were registered simultaneously and transmitted to the QUIPS online database [29]. Eligible patients had to fulfill the following conditions for inclusion: age above 18, answering the questionnaire on the first postsurgical day after CS and informed written consent. Patients without sufficient knowledge of the German language were excluded. Requirements were met in all participating departments and ethical approvals were available for each center. The study was approved by the principal investigators’ ethics committee (ethics committee of Bonn University Hospital, Registration Number: 208/18, ethics committee of Jena University Hospital, Registration Number 2772-12/09).

Distinction between PCS and UCS was performed using the German coding of operation and procedure classification system (OPS).

### Statistical Analysis

Fisher’s exact test or Chi squared was used as appropriate to analyze categorial data, presented as OR with 95% CI or percentage. Regarding continuous data, Kruskal–Wallis test, *t* test and Mann–Whitney U-test were performed and presented as mean plus/minus standard deviation (SD). Statistical significance was considered if the *p*-value was <0.05. SPSS version 19.0 (IBM SPSS Statistics for Windows, IBM Corp., Chicago, IL, USA) was used for statistical analysis. Multivariate logistic regression was used for the combined evaluation of variables that were shown to be significant in the Chi-squared test.

Moderate to severe pain was defined if the maximal pain intensity score was 5 or more. Severe pain was defined as 7 or more on the NRS. These cutoff points are widely used [1,31,32,33,34].

## 3. Results

**Figure 1:**
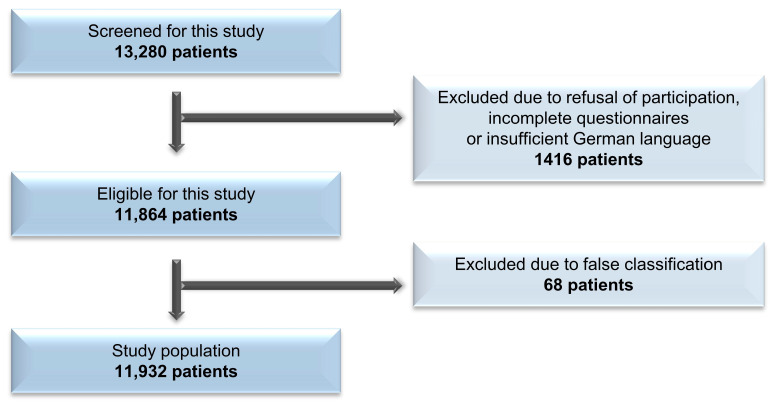
Selection process.

In total, 27 hospitals all over Germany met the inclusion criteria, and we screened 13,280 patients for this study; 1484 patients had to be excluded (reasons given in Figure 1). In total, data from 11,932 patients were evaluated for this study. Demographic characteristics can be found in Table 1.

### 3.1. Postoperative Pain Intensity

The mean value of maximal pain after surgery was NRS 6.49 (SD ± 2.09), the median NRS being 7.0 (interquartile range (IQR) 5.0–8.0). Overall, 82.2% of the patients reported moderate to severe pain with NRS ≥ 5, while 53.9% of the women suffered severe pain with NRS ≥ 7. Patients who suffered from chronic pain before CS had severe pain after CS significantly more often (68.5%) than those without a history of chronic pain (53.3%, *p* < 0.001). The wish for more analgesia was significantly higher in patients who reported severe pain with an OR of 3.60 (CI 3.17–4.09). Women who reported severe pain after CS reported significant lower satisfaction levels (*p* < 0.001). The mean satisfaction score (NRS 0–10, 0 being completely dissatisfied and 10 very satisfied) regarding analgesic treatment in the group of severe pain was 7.72 (SD ± 1.98), and in the group without severe pain it was 8.55 (SD ± 1.66) (*p* < 0.001).

### 3.2. Impact of Severe Pain

**Figure 2:**
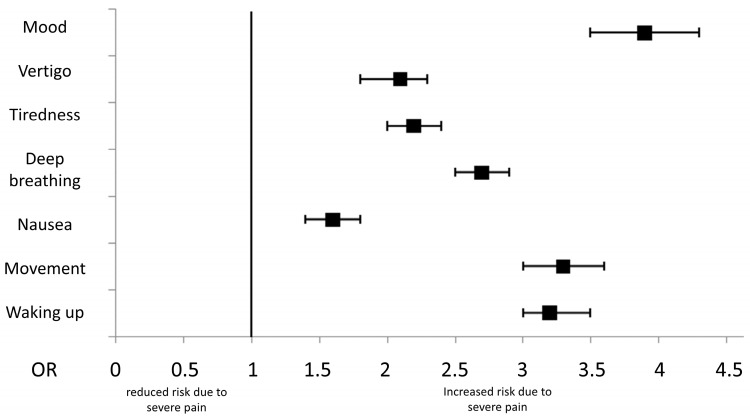
Negative impact of severe pain on daily life activities (NRS ≥ 7); black square indicates odds ratio (OR) and brackets indicate 95% confidence interval (CI).

Noticeable negative impacts of high pain levels on several daily life activities could be demonstrated (Figure 2). Patients reporting severe pain were more likely to report significant impairments of mood (30.9% vs. 10.4%, OR 3.85, CI 3.48–4.27), interference with deep breathing and coughing (80.9% vs. 61.1% OR 2.71, CI 2.49–2.94) and with ambulation (88.6% vs. 70.1%, OR 3.31, CI 3.00–3.64). Severe pain was also significantly related to postoperative symptoms like vertigo (29.0% vs. 16.6%, OR 2.06, CI 1.84–2.32), tiredness (53.4% and 34.3%, OR of 2.20, CI 2.04–2.37) and nausea (16.8% vs. 11.3%, OR 1.59, CI 1.43–1.77). Waking up in the first postoperative night due to pain was also significantly more often reported in the group of severe postoperative pain, with 55.7 vs. 27.9% (OR 3.25, CI 3.01–3.51, *p* < 0.001).

### 3.3. Analgesic Therapy

**Figure 3:**
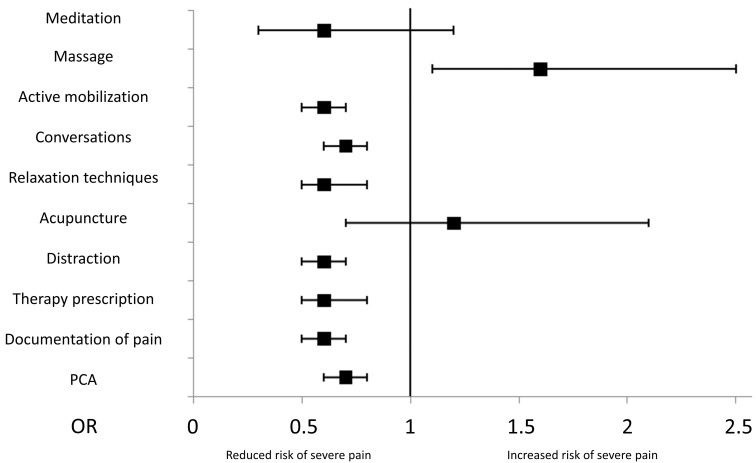
Influence of pharmacological and non-pharmacological pain therapy on severe pain, black square indicates odds ratio (OR) and brackets indicate 95% confidence interval (CI). PCA: patient-controlled analgesia.

Analgesic management was associated with pain intensity in different directions. Patients who complained about severe pain received slightly, but statistically significantly, more often opioids (41.9% vs. 40.1% (*p* < 0.05)). However, opioid-based patient-controlled analgesia (PCA) was significantly less widely used in patients reporting severe pain (10.1% vs. 14.3% (*p* < 0.001)), and this treatment resulted to be protective against severe pain (OR 0.67, CI 0.60–0.75, (*p* < 0.001, Figure 3)). Furthermore, it had a significant positive impact on pain-related impairment of sleep, deep breathing, movement, tiredness and mood (*p* < 0.001), without any influence on nausea or dizziness. Overall, it was only used in 12.1% of the cases.

Furthermore, receiving non-opioid analgesics was associated with a lower risk of developing severe pain, but this difference was very small (53.0% vs. 55.1%, OR 0.92, CI 0.86–0.99, *p* < 0.05). Local anesthesia for wound infiltration was used in only 6.4%, and the influence on pain severity could not be demonstrated (*p* > 0.05). Documentation of pain levels seemed to have a significant protective impact on developing severe pain. If a documentation of pain in clinical routine was present, significantly fewer patients reported severe pain compared to patients wherein no pain score was documented in the medical records (53.3% vs. 66.2%, OR 0.58, CI 0.50–0.68). Furthermore, the presence of therapy prescription for on-demand medication also seemed to have a significant positive impact on severe pain events. In total, 54.2% of women with individual therapy prescriptions suffered from severe pain, whereas 65.2% of those without documented treatment prescription reported NRS scores of 7 and above (*p* < 0.05, OR 0.63, CI 0.47–0.84, Figure 3). Time of surgery—whether during the day or night—had no influence on pain levels (*p* > 0.05).

### 3.4. Non-Pharmacological Therapy

Distraction in the form of reading or watching television was associated with a lower risk of reporting severe pain (OR 0.58, CI 0.50–0.68); it was used by 56.5% of all patients. Relaxation techniques were practiced by 12.2% of women after surgery, showing a significant positive effect on severe pain (OR 0.64, CI 0.51–0.80). Having conversations was significantly related to less severe pain. Dialogues with medical staff (performed in 45.2%) as well as with non-medical visitors like friends or relatives (carried out in 62.0%) also proved to be protective against severe pain (OR 0.71, CI 0.61–0.82 and OR 0.65, CI 0.56–0.75, respectively). Active mobilization in order to reduce pain was conducted in 38.6%, and showed a protective effect regarding severe pain (OR 0.56, CI 0.47–0.68, Figure 3). Distraction, relaxation, active mobilization and conversations with visitors as well as medical staff also had a significant positive impact on wish for more analgesia (*p* < 0.05). For some non-medical practices, the number of users was not sufficient to analyze any effect on severe pain, as can be seen in Figure 3, due to the wide CI. This was the case for TENS (7 users), mediation and praying (38 and 40 users), or massage (109 users) and acupuncture (53 users).

### 3.5. Primary vs. Urgent/Emergency Cesarean Section

In total, 7686 patients (64.4%) undergoing PSC and 4246 (35.6%) after UCS were investigated. Preexisting chronic pain was low in both groups and equally distributed, with 3.2% in PCS and 2.8% in UCS. Patients with UCS underwent general anesthesia significantly more often, whereas women with PCS received regional anesthesia more often (*p* < 0.001). Regarding the duration of surgery, it was slightly, but significantly, longer in PCS (mean 40.2 min (SD ± 19.6)) than in UCS (mean 39.0 min (SD ± 20.5), *p* < 0.001). Clinical data as well as demographic characteristics are described in Table 1.

There are no significant differences in the mean value of maximal pain, this being 6.48 (SD ± 2.12) in PCS and 6.50 (SD ± 2.01) in UCS (*p* > 0.05). Also, the distribution of severe pain (53.8% vs. 54.1%) and moderate to severe pain (81.6% vs. 83.0%. (*p* > 0.05)) was similar between PCS und UCS. Satisfaction with analgesic therapy did not differ statistically (*p* > 0.05). After adjusting for the presence of chronical pain, pain therapy, prescription of analgesics, regular pain assessment and type of anesthesia, the risk of severe pain was significantly higher in patients after UCS (OR 1.2, CI 1.1–1.3). Furthermore, a significantly higher number of patients complained about the impairment of movement due to pain in the group of UCS, with 82.0%, compared to the group of PCS, with 78.4% (OR 1.26, CI 1.14–1.39, *p* < 0.001). Pain-related impairment of deep breathing were also significantly more common in patients who underwent UCS (73.1%) in comparison to PCS (70.5%, OR 1.14, CI 1.04–1.24, *p* < 0.05). Furthermore, the consumption of opioids as well as non-opioids was significantly higher in women after UCS than after PCS (*p* < 0.001). In total, 71.6% of patients after UCS received non-opioids, whereas patients after PCS obtained non-opioids in 53.7% of cases (OR 2.17, CI 2.00–2.36). Ibuprofen was used in UCS in 61.4% and in PCS in 47.8% of cases (*p* < 0.001, OR 1.74, CI 1.60–1.88). Diclofenac was used rarely in both groups, but significantly more often in women after UCS (5.9% vs. 3.0%, respectively, *p* < 0.001). Also, metamizole was used more often after UCS, with 5.1%, compared to 4.2% after PCS (*p* < 0.05). The utilization of paracetamol was recorded in 38.2% of patients who underwent UCS and in 20.3% after PCS (*p* < 0.001). Opioids were used in 46.1% of patients after UCS and in 40.9% after PCS (OR 1.24, CI 1.14–1.34, *p* < 0.001). The opioid profiles between the two groups were different in terms of the types of prescribed opioid (see Table 2). Moreover, the utilization of PCA was significantly different between the two groups. Patients after UCS used PCA in 20.0% of cases, whereas women after PCS only used it in 7.3% of cases (OR 3.17, CI 2.80–3.59, *p* < 0.001).

## 4. Discussion

To our knowledge, with 11,932 evaluated patients, this is the largest prospective, multi-center cohort study so far to analyze the quality of postoperative analgesic care after CS using PROs under daily care conditions. Alarmingly high pain levels after CS can be observed. More than half of all investigated women suffered severe pain levels after CS. Those patients with severe pain events wished for more analgesia over three times more often, and they were significantly less satisfied with analgesic treatment in comparison to women without severe pain events.

In this research, median of maximal postoperative pain was NRS 7.0 (IQR 5.0–8.0) and the mean value was NRS 6.49 (SD ± 2.09). This is in line with the findings of Jiménez Cruz et al., who evaluated 409 patients regarding postoperative pain. They showed a mean value of NRS 6.98 (SD ± 2.08) and ranked CS as the most painful gynecological surgery [1]. Furthermore, Marcus et al. postulated that the median of maximal postoperative pain after CS was NRS 6.2 (CI 5.9–6.4) in the 811 patients they analyzed undergoing CS, in contrast to maximal pain intensity after hysterectomy in 2406 patients [35]. Gerbershagen et al. found CS to be 9th most painful operation out of 179 different surgeries. The mean value of maximal pain in that study was NRS 6.14; having evaluated 818 patients after CS [2]. A risk factor for developing severe pain was preexisting chronic pain, with an OR of 1.90 (CI 1.52–2.38). This correlation has been reported before [1,35].

The inevitable question arises as to why patients after CS are suffering that amount of pain, and why no change has occurred despite the deficiency having been known of for years. One potential cause could lie in the widespread assumption of pain being natural in the birthing experience [14,15,16,17]. According to McCauley et al., half of all healthcare providers do not routinely prescribe medication for pain relief after birthing, since they consider labor pain as necessary for birth [36]. Hence, although CS is a surgery with major tissue trauma, it falls into the obstetric category and is therefore likely to be treated differently compared to pain therapy. Another reason for insufficient pain treatment could be the hesitation of medical staff to prescribe opioids in breastfeeding mothers because of the assumed side effects for newborns. Also, this concern arises in patients as well [25]. In the study of Carvalho et al., pregnant women claimed to withstand pain of 56 ± 22 on a visual analog pain score (0–100 mm) before considering taking any pain medication due to worries of harming the newborn. The women stated in the same evaluation that fear of pain was their highest concern regarding the postoperative time span after CS [37]. Since in a recent study, the wish for more analgesia was over three times higher in patients suffering severe pain, it is to be assumed that the low amount of opioids taken is not only due to patients’ restraint. It could be demonstrated that only 41.9% of patients having severe pain events were treated with opioids. The utilization of opioids in addition to non-opioid therapy is recommended in the NICE (National Institute for Health and Care Excellence) guidelines for cesarean birth [38], as well as by the PROSPECT (procedure specific postoperative pain management) Working Group of the European Society of Regional Anesthesia and Pain Therapy [39], the German guidelines of analgesia and anesthesia in obstetrics [40] as well as in the German guidelines for therapy of acute perioperative posttraumatic pain [41]. Due to its excellent and prolonged (14 to 36 h) postoperative analgesia, intrathecally applied morphine is the gold standard in analgesic treatment after CS, according to Sutton et al. [42]. It is also recommended by the NICE guidelines [38], the American practice guidelines for obstetric anesthesia [43] and the German guidelines for therapy of acute perioperative posttraumatic pain [41]. Recent studies investigated the spinal administration of morphine in comparison to fentanyl using PROs for postoperative pain management after CS. They demonstrated a positive effect of intrathecal morphine regarding required rescue medication after surgery [44,45]. Also, a prolonged analgesic effect could be seen in a recent meta-analysis by La Via et al. in relation to the utilization of α2-agonists in spinal anesthesia for CS compared to fentanyl, which may contribute to reduce shivering, nausea or vomiting [46].

Furthermore, NICE guidelines recommend offering women oral, intravenous or subcutaneous morphine after CS; they state that there usually is no impact on breastfeeding newborns. If the patient needs stronger pain medication, oxycodone or tramadol can be considered. When using these opioids in breastfeeding mothers, patient information regarding the possible risk of neonatal sedation and respiratory depression should be collected. If the patient is receiving opioids, the prescription of laxatives should be considered for the prevention of constipation. Codeine should not be offered to breastfeeding women because of neonatal sedation [38]. Therefore, the education of patients as well as medical staff regarding opioids and their safety in breastfeeding newborns should be emphasized to encourage their usage in pain reduction after CS.

PCA after CS is recommended in the NICE guidelines [38] and German guidelines [25]. If no epidural was applied, intravenous application is suggested [38]. According to Marcus et al., patients who have been offered PCA used opioids in higher amounts than those without. They assume this was due to the bypassing of insufficient nurse-controlled opioid administration [35]. In this study, PCA was found to be used in only 12.1% of all patients. Including PCA in standard postoperative treatment could help improve pain management after CS, especially if CS is performed under general anesthesia when there is no protective effect of regional anesthesia. Regarding non-opioids, NICE recommends using a combination of paracetamol and ibuprofen, and they suggested regular administration instead of on-demand application for pain relief. Also, the American Pain Society and German guidelines recommend a multimodal concept of pain treatment with around the clock non-opioid therapy [5,38,40]. Astonishingly, this study revealed a markedly low utilization of non-opioids in patients after CS. Only 59.0% of all patients received non-opioids. Those women enduring severe pain levels only received non-opioid medication in 53.0% of cases. Taking the recommendations of international guidelines into consideration, major improvements in prescribing non-opioids as the basis of pain treatment after CS could be achieved.

This study shows low rates of local anesthesia use for wound infiltration, at only 6.4% in total, and no difference regarding severe pain levels could be registered following the usage of wound infiltration. Sutton et al. rates the utilization of local anesthesia in the context of a multimodal analgesia model as an important tool for pain reduction after CS [42]. Its application is recommended by the German guidelines for acute perioperative posttraumatic pain, especially if no intrathecal opioids have been used [41]. Several studies have concluded that there is a positive effect of wound infiltration on postoperative pain after CS [47,48,49]. This discordance of results is assumed to be due to the small number of patients having received wound infiltration in this study, and probably due to the heterogeneity of the application technique. The combined application of subfascial and epifascial treatments seems to offer the best analgetic profile in these cases.

In this research, no significant difference was seen in pain relief regarding the usage of TENS. However, only 0.2% (seven patients) of all patients used TENS, and therefore the validity of this result is low. In contrast to these results, several studies showed that the utilization of TENS had a significantly positive effect on pain after CS [50,51,52,53]. In a prospective, randomized study, Kayman-Kose et al. evaluated 50 patients who were treated with TENS; they showed significantly lower pain scores (*p* < 0.001) in comparison to placebo. The electrodes were placed above and below the incision line and were applied once after CS for 30 min [50]. The use of TENS after CS is also recommended by the American Pain Society [5] and German guidelines for acute perioperative posttraumatic pain [41]. The difference of outcomes from those in other research can be assumed to be due to the insufficient numbers of patients using TENS in the present study, or the deficient application of TENS. Based on several publications pointing out the positive effects on postoperative pain after CS, TENS should be implemented in standard care, as it is a noninvasive, non-pharmacological method without any side effects.

As far as we know, this is the first study investigating several non-pharmacological measures that can be performed by the patient to reduce postoperative pain after CS. Interestingly, these simple methods can positively influence the postoperative pain experience significantly. We were able to show that distraction, for example in the form of reading or watching television, relaxation techniques, and having conversations with family or medical staff were associated with significantly reduced risks of severe pain and desire for more analgesia. Furthermore, active mobilization had a significant impact on postoperative pain. Some authors showed the significant effects of praying, meditation and acupuncture [54,55,56]; these results could not be supported by the present data due to the small amounts of people using these techniques. The American Pain Society recommends multimodal pain management, including the use of non-pharmacological tools [5]. Altogether, non-medical methods potentially significantly affect the postoperative pain after CS. Their implementation in pain treatment should be considered, since these methods are easy to use and cost-effective. Educating patients concerning those measures could improve relieving pain, reduce analgesics and enhance patient recovery.

Simple pain assessments executed by medical staff were found to be significantly associated with improved postsurgical pain after CS. The availability of written therapy prescriptions and regular documentation of pain scores in patient records were protective against severe pain. To our knowledge, these parameters and their effects on pain after CS have not been evaluated so far. The relation to severe pain of these measures seems obvious though. Departments monitoring pain scores after surgery and providing written analgesic prescription normally have internal protocols for postoperative analgesia, so therapy is standardized, and the clinic staff have experience in managing high pain levels [57]. Also, having some kind of monitoring system for pain allows staff to identify patients in pain before their pain levels are too high. Including these simple tools in daily care can improve pain treatment with little effort.

It has been shown before that the quality of analgesic therapy, as well as postoperative recovery, depends on multidimensional factors, such that evaluating pain intensity as the only factor is insufficient in measuring those outcomes [35,58,59,60]. Therefore, in this study the pain-related impact on daily life activities was analyzed using PROs. A significant negative influence of severe pain on mood, movement, tiredness, deep breathing, night sleep as well as vertigo and nausea could be demonstrated. Since patients with severe pain used significantly more opioids, the vertigo and nausea could be a side effect of opioid therapy. The correlation between high pain scores and delayed patient recovery after surgeries has been demonstrated before [61,62,63]. Marcus et al. compared postoperative pain after CS with pain after hysterectomies. They revealed more pain-related impairments of movement and deep breathing in patients undergoing CS than hysterectomy, although both interventions were comparable in terms of invasiveness and thus expected pain intensity [35]. The findings of this study are also in line with the results of Jiménez Cruz et al. They found significant impairments of mobilization, deep breathing, mood and nightsleep, as well as enhanced tiredness, nausea and dizziness, due to moderate to severe pain (NRS > 5) after gynecological surgeries [1].

Regarding the comparison of pain-related outcomes between PCS and UCS, no difference concerning pain levels could be demonstrated. Concerning maximal pain after CS, surgery was shown to have a significant influence on severe pain in the multivariate analysis. However, a significant difference in pain-related movement and deep breathing in favor of PCS could be shown. Furthermore, significantly more opioids as well as non-opioids were administered to patients after UCS. The time point of surgery—whether during day or night—had no influence on pain levels; these findings are in contrast to those of Jiménez Cruz et al. [1]. These differences could be due to a larger and multicentric sample size in this present study. Therefore, this being caused by worse pain treatment during night shifts is unlikely. Furthermore, there was no influence of the type of anesthesia on pain levels after CS. So, although UCSs were more often performed under general anesthesia, it is unlikely that this is the cause of the greater analgesia- and pain-related impairments of daily life activities resulting from UCS.

The importance of adequate pain treatment after CS has attracted interest in international guidelines. The American Pain Society recommends informing pregnant women about different possibilities of postoperative pain treatment early on when planning a CS [5]. German guidelines acknowledge the insufficient pain treatment of patients after CS in Germany, and advocate improving analgesic therapy, including offering opioids as standard procedures [25]. To help achieve that goal, the benchmarking tools QUIPS and its international version PAIN OUT can be employed. Thereby, an inter-clinical comparison between participating departments is enabled, and self-reflection as well as assessment can be encouraged [1,2,34].

## 5. Limitations

The limitations of this study include the voluntary participation resulting in a possible selection bias. In order to minimize this effect, a multi-center approach including 27 hospitals was employed. Since the exclusion of patients without sufficient knowledge of the German language was enacted, the investigated population may have been too unilateral. There was no possibility to evaluate sociocultural influences. The lack of assessment of psychological aspects could be considered a limitation of this study. Other authors have shown that psychologic aspects such as anxiety, catastrophizing or depression levels could have an influence on postoperative pain scores [64,65,66]. Another possible limitation to be mentioned may be the heterogeneity of the evaluated PROs and the wide range of different managements addressed in this study. This makes the interpretation of the presented results regarding causality limited. This was expected at the time of the study’s design. In order to minimize this possible confounder, a large sample size was evaluated, but this led to heterogeneity, meaning this manuscript probably only reflects the present German scenario.

Furthermore, significant differences may not always be clinically relevant, since a great number of patients were evaluated. Moreover, the ultimate effects of different surgical techniques have not been taken into consideration. This topic should be investigated in further research.

As it is impossible to control for all covariables, associations found in non-randomized observational data such as those in the QUIPS registry should be carefully interpreted, and may not constitute a causal relation.

## 6. Conclusions

This study shows substantial shortcomings in current practices of pain management after CS. More than half of all patients are suffering severe pain after this surgery. The implementation of sufficient analgesic and non-analgesic pain management is of the utmost priority. In particular, the education of medical staff as well as patients regarding the safety of using certain opioids in breastfeeding mothers should be undertaken. Severe pain has a major effect on daily life activities and recovery after CS. Its adequate treatment should be an integral component of quality of care.

## Figures and Tables

**Table 1:** Demographic characteristics. CS: cesarean section; PCS: primary cesarean section; UCS: urgent/emergency cesarean section; n: number; SD: standard deviation.

	CS n = 11,932	PCS n = 7686	UCS n = 4246
	n (%)	n (%)	n (%)
Age (years)			
18–20	244 (2.1)	146 (1.9)	98 (2.3)
21–30	4546 (38.3)	2786 (36.2)	1760 (41.5)
31–40	6432 (54.9)	4225 (55.0)	2207 (52.0)
≥41	478 (4.0)	332 (4.3)	146 (3.4)
Missing data	232 (1.9)	197 (2.6)	35 (0.8)
Preoperative chronic pain			
No pain	11,466 (96.9)	7360 (95.8)	4106 (96.7)
Pain	363 (3.1)	244 (3.2)	119 (2.8)
Missing data	103 (0.9)	82 (1.1)	21 (0.5)
Type of anesthesia			
General	996 (8.3)	494 (6.4)	502 (11.8)
Regional	8580 (71.9)	5434 (70.7)	3146 (74.1)
Missing data	2356 (19.7)	1758 (22.9)	598 (14.1)
Duration of surgery (min)	Mean 39.8 SD 20.5	Mean 40.2 SD 19.6	Mean 39.0 SD 21.7

**Table 2:** Distribution regarding utilization of opioids in PCS and UCS. PCS: primary cesarean section; UCS: urgent/emergency cesarean section; OR: odds ratio; CI: confidence interval; n: number.

	PCS	UCS	OR (CI)
	n (%)	n (%)	
Opioid	2484 (40.9)	1920 (46.1)	1.24 (1.14–1.34)
Oxycodone	1528 (25.1)	767 (18.4)	0.67 (0.61–0.74)
Morphine	122 (2.0)	135 (3.2)	1.64 (1.28–2.10)
Pethidine	65 (1.1)	94 (2.3)	2.14 (1.55–2.94)
Piritramide	955 (15.7)	988 (23.7)	1.67 (1.51–1.84)
Sufentanil	48 (0.8)	53 (1.3)	1.62 (1.09–2.40)
Tramadol	215 (3.5)	228 (5.5)	1.58 (1.31–1.91)
Missing information about indication for surgery 1692 (14.2)			

## Data Availability

The data underlying this article are available in the article and in its online supplementary material.

## Supplementary Materials

The following supporting information can be downloaded at: https://www.mdpi.com/article/10.3390/jcm12226999/s1, Supplementary S1: Translation of German questionnaire for QUIPS.

## Abbreviations

Cesarean section	CS
Confidence interval	CI
Interquartile range	IQR
Numeric rating scale	NRS
Odds ratio	OR
Primary cesarean section	PCS
Quality improvement in postoperative pain management project	QUIPS
Standard deviation	SD
Urgent/emergency cesarean section	UCS
