# Supplementary material for: Risk Factors for Severe Pain and Impairment of Daily Life Activities after Cesarean Section—A Prospective Multi-Center Study of 11,932 Patients
Source: J Clin Med. 2023 Nov 9;12(22):6999. doi: 10.3390/jcm12226999 (PMC10672043; doi:10.3390/jcm12226999)
Supplement: Supplementary file 1 [file jcm-12-06999-s001.zip › jcm-2658001-supplementary.pdf]

Supplementary S1

QUIPS Result Questionnaire

Dear Patient,

now that you have decided to participate, we kindly ask you to fill in the questionnaire. Thank you very much.

The following questions are about your **pain since your surgery.**

1. How severe has your pain been since surgery when you have been straining**,** for example, **mobilizing, moving, washing, coughing, breathing through?** Please mark the number on the scale that applies to your pain on exertion ("0" means no pain and "10" means the strongest pain imaginable).

0	1	2	3	4	5	6	7	8	9	10

No pain — Strongest pain imaginable

2. Please check off the **most severe pain** you have had since your surgery:

0	1	2	3	4	5	6	7	8	9	10

No pain — Strongest pain imaginable

3. Please check here the **least pain** you have had since your surgery:

0	1	2	3	4	5	6	7	8	9	10

No pain — Strongest pain imaginable

The next four questions are about whether certain activities or your mood have been **affected by pain** since surgery.
By "impaired" is meant: the activity is **impossible** or **possible only with great difficulty**.

Are you impaired by the pain:

4. in your ability to move / ambulate?
❑ Yes ❑ No

5. when **coughing or taking a deep breath?**
❑ Yes ❑ No

6. while **sleeping?**
❑ Yes ❑ No

7. in your **mood?**
❑ Yes ❑ No

8. Have you felt very **tired** since the operation?
❑ Yes ❑ No

9. Have you suffered from **nausea** since the operation?
❑ Yes ❑ No

10. Have you suffered from **dizziness** since the operation?
❑ Yes ❑ No

Copyright Winfried Meißner/ University Hospital Jena. Use by third parties subject to approval by DGAI/ DGCH Version 3.2

11. Have you been informed about the different **options available to you for your pain management?**

❑ Yes ❑ No

12. Were you involved as much as you wanted about your **pain therapy?**

?

0	1	2	3	4	5	6	7	8	9	10

Not involved at all Totaly involved

13. Would you have wished to have MORE pain medikation than you received?

❑ Yes ❑ No

14. Please tick how **satisfied** you are with the result of your **Pain management** since your surgery:

0	1	2	3	4	5	6	7	8	9	10

Completely dissatisfied Very satisfied

15. Have you used or received **non-drug** methods for **pain relief**?

❑ Yes ❑ No If

yes, which?

❑ Cooling compresses ❑ Heat ❑ Meditation ❑ Deep breathing

❑ Acupuncture ❑ Pray ❑ Walk around ❑ Massage

❑ Distraction (e.g. watching TV, reading) ❑ Imagination ❑ Relaxation ❑ TENS (Transcutaneous electrical nerve stimulation)

❑ Discussions with medical staff ❑ Conversations with friends/ relatives

16. Did you have **constant pain** that lasted **three months or more** before you came to the hospital for this surgery?

❑ Yes ❑ No

a. If so, **how severe** was this pain most of the time?
Please check the value that best reflects your pain intensity.

0	1	2	3	4	5	6	7	8	9	10

No pain Strongest pain imaginable

b. If so, **where did** this **constant pain occur**?

❑ on the part of the body that was operated on
❑ on another part of the body
❑ both (surgical site and elsewhere)

Copyright Winfried Meißner/ University Hospital Jena. Use by third parties subject to approval by DGAI/ DGCH Version 3.2

To be completed by interviewer: Patient was interviewed: ❑ Yes ❑ No

Copyright Winfried Meißner/ University Hospital Jena. Use by third parties subject to approval by DGAI/ DGCH Version 3.2
